# Clinical Characteristics of Children with Autism Spectrum Disorder and Co-Occurring Epilepsy

**DOI:** 10.1371/journal.pone.0067797

**Published:** 2013-07-04

**Authors:** Emma W. Viscidi, Elizabeth W. Triche, Matthew F. Pescosolido, Rebecca L. McLean, Robert M. Joseph, Sarah J. Spence, Eric M. Morrow

**Affiliations:** 1 Department of Epidemiology, Division of Biology and Medicine, Brown University, Providence, Rhode Island, United States of America; 2 Department of Molecular Biology, Cell Biology and Biochemistry and Institute for Brain Science, Brown University, Laboratory for Molecular Medicine, Providence, Rhode Island, United States of America; 3 Developmental Disorders Genetics Research Program, Emma Pendleton Bradley Hospital and Department of Psychiatry and Human Behavior, Brown University Medical School, Providence, Rhode Island, United States of America; 4 Neurodevelopmental Center, Department of Pediatrics, Memorial Hospital of Rhode Island, Brown University Medical School, Pawtucket, Rhode Island, United States of America; 5 Department of Anatomy and Neurobiology, Boston University School of Medicine, Boston, Massachusetts, United States of America; 6 The Autism Consortium, Boston, Massachusetts, United States of America; 7 Department of Neurology, Boston Children’s Hospital, Harvard Medical School, Boston, Massachusetts, United States of America; Dartmouth Medical School, United States of America

## Abstract

**Objectives:**

To estimate the prevalence of epilepsy in children with Autism Spectrum Disorder (ASD) and to determine the demographic and clinical characteristics of children with ASD and epilepsy in a large patient population.

**Methods:**

Cross-sectional study using four samples of children with ASD for a total of 5,815 participants with ASD. The prevalence of epilepsy was estimated from a population-based sample. Children with and without epilepsy were compared on demographic and clinical characteristics. Multivariate logistic regression was used to examine the association between demographic and clinical characteristics and epilepsy.

**Results:**

The average prevalence of epilepsy in children with ASD 2–17 years was 12.5%; among children aged 13 years and older, 26% had epilepsy. Epilepsy was associated with older age, lower cognitive ability, poorer adaptive and language functioning, a history of developmental regression and more severe ASD symptoms. The association between epilepsy and the majority of these characteristics appears to be driven by the lower IQ of participants with epilepsy. In a multivariate regression model, only age and cognitive ability were independently associated with epilepsy. Children age 10 or older had 2.35 times the odds of being diagnosed with epilepsy (p<.001) and for a one standard deviation increase in IQ, the odds of having epilepsy decreased by 47% (p<.001).

**Conclusion:**

This is among the largest studies to date of patients with ASD and co-occurring epilepsy. Based on a representative sample of children with ASD, the average prevalence of epilepsy is approximately 12% and reaches 26% by adolescence. Independent associations were found between epilepsy and older age and lower cognitive ability. Other risk factors, such as poor language and developmental regression, are not associated with epilepsy after controlling for IQ. These findings can help guide prognosis and alert clinicians to patients with ASD who are at increased risk for epilepsy.

## Introduction

Autism spectrum disorder (ASD) is a neurodevelopmental disorder characterized by deficits in social interaction and communication and the presence of restricted and repetitive behavior. Epilepsy is a neurologic condition characterized by recurrent, unprovoked seizures [1]. The co-occurrence of ASD and epilepsy is well established among clinicians and researchers [2], [3] but the characteristics of children with both conditions have not been studied in large, contemporary datasets. Epilepsy is commonly reported to occur in 30% of individuals with ASD [3], [4], [5], [6], which exceeds that of the general population (0.7–1%) [7] but prevalence estimates have varied widely, ranging from 5% to 46% [2], [3], [8], [9], [10], [11], [12], [13], [14], [15], [16], [17]. This variation is likely due to differences between prior studies in the age and cognitive level of participants and in the sampling and diagnostic methods used. Many previous studies of epilepsy in ASD have had small sample sizes that are unlikely to be representative of the general ASD population and insufficiently powered to make rigorous conclusions about risk factors [8],[18]. In addition, some prior studies were based on previous diagnostic criteria for ASD [10],[19].

Prior studies have reported that epilepsy in ASD is associated with female gender [15],[18],[20],[21], lower cognitive ability [9],[14],[15],[20],[21] and adaptive functioning [21], and a history of developmental regression [22],[23]. However, findings have been inconsistent and often contradictory, which is likely due to small sample sizes. At present, there is insufficient information to make strong predictions as to which individuals with ASD are at greatest risk for epilepsy and what the associated clinical characteristics may be.

We conducted among the largest studies to date on the co-occurrence of ASD and epilepsy. The aim was to compare children with ASD and epilepsy to children with ASD alone on demographic and clinical characteristics. Participants were drawn from four samples; an epidemiologic population-based sample (the 2007 National Survey of Children’s Health) and three genetic collaborative samples (the Autism Genetic Resource Exchange, the Simons Simplex Collection, and the Autism Consortium). Combining all samples, a total of 5,815 individuals with ASD were studied. Using this large sample we provide important insights regarding the prevalence and clinical correlates of epilepsy in this subgroup of patients with ASD.

## Methods

### Ethics Statement

All research was approved by institutional review boards (IRB). Parents gave informed consent to participate in each of the studies. For the 2007 NSCH study, the NSCH IRB approved all study procedures. Verbal informed consent for study participation was approved by the NSCH IRB. Written consent was not obtained because of the nature of the phone-based interview. Verbal consent was obtained and documented in the computer-assisted telephone interview (CATI) system. For the AGRE, SSC, and AC studies written consent was obtained according to procedure that was approved by the ethics committees. Research was approved by the Lifespan Health IRB Study # 4114–09.

### Subjects

Subjects were enrolled in one of the four studies described below. See **Table 1** for a summary of the study populations.

**Table 1:** Summary of Samples and Measures.

Sample	The Autism GeneticResource Exchange (AGRE)	The Simons Simplex Collection (SSC)	The AutismConsortium (AC)	The 2007 National Survey ofChildren’s Health (NSCH)
Total ASD Sample Size	2,524	1,891	479	921
Description of Sample	Majority multiplex families (morethan one child with ASD) in U.S.	Simplex families (only onechild with ASD) in U.S.	Simplex and multiplexfamilies in New England.	Population-based survey of children in U.S.
ASD Diagnosis	Standardized diagnostic assessments	Standardized diagnostic assessments	Standardized diagnostic assessments	NSCH survey
Epilepsy Diagnosis	ADI-R	ADI-R	ADI-R	NSCH survey
Cognitive Ability	Available	Available	Available	Not available
Adaptive Functioning	Available	Available	Available	Not available
Language	Available	Available	Available	Not available
Developmental Regression	Available	Available	Available	Not available
ASD Severity	Available	Available	Available	Not available

Abbreviations: ASD, Autism Spectrum Disorder; ADI-R, Autism Diagnostic Interview–Revised.

### 2007 National Survey of Children’s Health (NSCH)

The NSCH is a nationally representative random-digit-dial telephone-based survey sponsored by the U.S. Department of Health and Human Services Administration Maternal and Child Health Bureau and conducted by the National Center for Health Statistics of the Centers for Disease Control and Prevention. A parent/guardian was asked questions regarding the child’s health during a telephone interview. A sampling weight was provided by the NSCH with the data record for each child. This weight is based on the probability of selection of the child’s telephone number, with adjustments for known survey response biases and further adjustments to ensure that weighted estimates match demographic control totals from the U.S. Census Bureau’s American Community Survey. Weighted results represent the population of non-institutionalized children ages 0–17 at the national and state level. Substantive and methodological details of the survey have been previously described [24]. Out of 81,176 children aged 2 to 17 years in the sample, there were 921 children with a current diagnosis of ASD used in the present study.

### Autism Genetic Resource Exchange (AGRE)

The AGRE is a collection of genetic and phenotypic data on families with ASD from across the United States. The majority of families have more than one child affected with ASD (multiplex families) [25],[26]. Detailed information on the study methodology has been previously described [26]. The AGRE sample used in the present study includes 2,524 individuals with ASD.

### Simons Simplex Collection (SSC)

The SSC is a collection of genetic and phenotypic data on simplex families (one child affected with ASD) across the United States. Families were recruited from clinics serving children with ASD and were included if the family had only one child aged 4–18 years who met criteria for ASD. Detailed information on inclusion and exclusion criteria can be found in the SFARI Base/SSC Researcher Welcome Packet [27] and additional information on the study methodology has been previously described [28]. The SSC sample used in the present study includes 1,891 children with ASD from version 9 (released 8/2/2010).

### Autism Consortium (AC)

The AC is a collection of genetic and phenotypic data on simplex and multiplex families of individuals with ASD in the Massachusetts area. Families were recruited from Boston area hospitals. There were no specific inclusion or exclusion criteria. The AC sample used in the present study includes 479 individuals with ASD.

### Measures

#### Autism Spectrum Disorder (ASD)

All of the subjects in the study had been diagnosed, or were reported to be diagnosed, with ASD. In the genetic collaborative samples, diagnosis of ASD was based on standardized diagnostic assessments including the Autism Diagnostic Interview–Revised (ADI-R) [29] and the Autism Diagnostic Observation Schedule (ADOS) [30]. The SSC and AC samples used in the present study included individuals meeting criteria for a less severe diagnostic classification of ‘ASD’ on the ADI-R (equivalent to DSM-IV PDD-NOS [31]) based on modified cut-off scores widely used in ASD research [32]. In the AGRE sample, only individuals who met ADI-R criteria for autism were included because information on the ‘ASD’ classification was not available.

In the NSCH, diagnosis of ASD was based on parent-report. During the phone interview, parents of children aged 2–17 years were asked if they had ever been told by a doctor or other health care provider that their child had “autism, Asperger disorder, pervasive developmental disorder, or other autism spectrum disorder.” If parents responded affirmatively, they were then asked if their child currently had ASD. All 2–17 year old children reported to be currently diagnosed with ASD were included in the present study (n = 921).

#### Epilepsy

Diagnosis of epilepsy was based on parent report in all of the samples. In the genetic collaborative samples, epilepsy was measured by parent response to an ADI-R question asking if the child “has ever fainted or had a fit or seizure or convulsion?” The child was classified as having been diagnosed with epilepsy if the parent reported that the child had a definite diagnosis of epilepsy. The child was classified as having never been diagnosed with epilepsy if the parent reported the child had had no attacks or febrile convulsions only. Sixty-three children from AGRE and one child from SSC were missing data on epilepsy and were therefore excluded from the analyses. Children reported to have a “history of attacks that might be epileptic, but diagnosis not established” were excluded from the analyses to prevent misclassification (n = 323 participants total from AGRE, SSC, and AC). Table 2 presents demographic characteristics of the entire sample, including these 323 participants. Tables 3–6 and Tables S1–S9 in Supporting Information S1 present analyses excluding these participants.

**Table 2:** Demographic Characteristics of Individuals with Autism Spectrum Disorder by Study Sample.

	AGRE	SSC	AC		NSCH
	(n = 2524)	(n = 1891)	(n = 479)		(n = 921)
Characteristic		No. (%)		Characteristic	No.^b^ (Wt. %)
Gender				Gender	
Male	2026 (80.3)	1633 (86.4)	388 (81.0)	Male	753 (81.0)
Female	498 (19.7)	258 (13.6)	91 (19.0)	Female	168 (19.0)
Age (years)				Age (years)	
Under 4	295 (11.7)	0 (0.0)	40 (8.4)	2–3	46 (3.1)
4–6	861 (34.1)	682 (36.1)	171 (35.7)	4–7	253 (27.4)
7–9	693 (27.5)	548 (29.0)	117 (24.4)	8–11	236 (33.4)
10–12	338 (13.4)	368 (19.5)	72 (15.0)	12–14	200 (22.2)
13–18	264 (10.5)	293 (15.5)	73 (15.2)	15–17	186 (14.0)
Over 18	73 (2.9)	0 (0.0)	6 (1.3)	Over 18	N/A
Race				Race	
White	1781 (70.6)	1492 (78.9)	401 (85.9)	White, Non-Hispanic	664 (66.9)
African-American	49 (1.9)	69 (3.6)	10 (2.1)	Black, Non-Hispanic	57 (8.3)
Asian	62 (2.5)	70 (3.7)	11 (2.4)	Hispanic	94 (19.1)
More Than One Race	170 (6.7)	157 (8.3)	30 (6.4)	Other, Non-Hispanic	86 (5.6)
Other	12 (0.5)	83 (4.4)	2 (0.4)		–
Not Specified	450 (17.8)	20 (1.1)	13 (2.8)		–
Type of ASD^a^					
Autism	2524 (100.0)	1700 (89.9)	428 (89.4)		N/A
ASD	N/A	191 (10.1)	51 (10.7)		N/A
Intellectual Disability (ID)					
Non- ID (IQ>70)	344 (66.7)	1345 (71.2)	238 (84.7)		N/A
ID (IQ≤70)	172 (33.3)	545 (28.8)	43 (15.3)		N/A

Abbreviations: ASD, Autism Spectrum Disorder; AGRE, the Autism Genetic Resource Exchange; SSC, the Simons Simplex Collection; AC, the Autism Consortium; NSCH, the 2007 National Survey of Children’s Health; Wt. %, weighted percentage; N/A: not applicable or data not unavailable.

a Diagnosis based on the Autism Diagnostic Interview, Revised (ADI-R) cut-off scores for autism and ASD (Risi et al. 2006).

b Unweighted number of children.

Values may not add up to total due to missing data.

**Table 3:** Distribution of Epilepsy among Individuals with Autism Spectrum Disorder by Study Sample.

	Distribution of Epilepsy				Prevalence of Epilepsy	
	AGRE	SSC	AC	Combined^a^	NSCH	
	(n = 2273)	(n = 1786)	(n = 450)	(n = 4509)	(n = 918)	
	No. (%)				No.^b^ (Wt. %)	(Wt. 95% CI)
Never Diagnosed with Epilepsy	2153 (94.7)	1735 (97.1)	420 (93.3)	4308 (95.5)	830 (87.5)	
Diagnosed with Epilepsy	120 (5.3)	51 (2.9)	30 (6.7)	201 (4.5)	88 (12.5)	(10.4–14.7)

Abbreviations: AGRE, the Autism Genetic Resource Exchange; SSC, the Simons Simplex Collection; AC, the Autism Consortium; NSCH, the 2007 National Survey of Children’s Health; Wt. %, weighted percentage; CI, confidence interval.

a Genetic Collaborative Samples (AGRE, SSC, and AC) combined.

b Unweighted number of children.

**Table 4:** Epilepsy Diagnosis by Gender and Age among Individuals with Autism Spectrum Disorder.

	Combined Genetic Collaborative Sample^a^			NSCH				
	(n = 4509)			(n = 921)				
	Never Diagnosed with Epilepsy	Diagnosed with Epilepsy		Never Diagnosed with Epilepsy		Diagnosed with Epilepsy		
	No. (%)		p-value	No.^b^ (Wt. %)			(Wt. 95% CI)	p-value
Age (years)			<.001					0.009
6 or under	1824 (97.8)	41 (2.2)		209 (94.6)	15 (5.4)		(2.4–8.4)	
7–9	1218 (96.2)	48 (3.8)		179 (92.9)	18 (7.1)		(3.5–10.7)	
10–12	682 (93.8)	45 (6.2)		172 (89.6)	17 (10.4)		(6.0–14.8)	
13 or older	574 (89.6)	67 (10.5)		270 (73.8)	38 (26.2)		(21.2–31.1)	
Gender			<.001					0.81
Male	3586 (96.1)	147 (3.9)		688 (87.3)	62 (12.7)		(10.3–15.1)	
Female	719 (93.0)	54 (7.0)		142 (88.5)	26 (11.5)		(6.7–16.4)	
Gender by Age								
Male			<.001					<.001
6 or under	1496 (98.1)	29 (1.9)		175 (95.7)	10 (4.3)			
7–9	1020 (96.4)	38 (3.6)		148 (91.2)	14 (8.8)			
10–12	581 (95.1)	30 (4.9)		144 (89.7)	11 (10.3)			
13 or older	484 (90.6)	50 (9.4)		221 (70.8)	27 (29.2)			
Female			<.001					<.001
6 or under	328 (96.5)	12 (3.5)		34 (87.8)	5 (12.2)			
7–9	198 (95.2)	10 (4.8)		31 (98.8)	4 (1.2)			
10–12	101 (87.1)	15 (12.9)		28 (88.6)	6 (11.4)			
13 or older	90 (84.1)	17 (15.9)		49 (81.2)	11 (18.8)			

Abbreviations: NSCH, 2007 National Survey of Children’s Health; Wt. %, weighted percentage; CI, confidence interval.

a Genetic Collaborative Samples (AGRE, SSC, and AC) combined.

b Unweighted number of children.

**Table 5:** Epilepsy Diagnosis by Clinical Characteristics among Individuals with Autism Spectrum Disorder, Genetic Collaborative Samples.

	Combined Genetic Collaborative Sample^a^		
	(n = 4509)		
	Never Diagnosed with Epilepsy	Diagnosed with Epilepsy	
	No. (%)		p-value
**Regression**			
Any Regression			<.001
No Definite Loss	3095 (96.4)	114 (3.6)	
Definite Loss	1210 (93.3)	87 (6.7)	
Loss of Any Language			.001
No	2911 (96.8)	96 (3.2)	
Yes	615 (94.2)	38 (5.8)	
Loss of Skills			<.001
No Consistent Loss	2680 (97.0)	82 (3.0)	
Probable Loss	127 (92.0)	11 (8.0)	
Definite Loss	712 (94.7)	40 (5.3)	
**Language**			<.001
Overall Level of Language			
Meaningful Use of Phrases	3366 (96.5)	123 (3.5)	
Fewer than 5 Words	463 (92.2)	39 (7.8)	
PPVT Score	3251 (85.9, 27.9)	110 (67.8, 31.3)	<.001
**Cognitive Ability**			
Intellectual Disability (ID)			<.001
Non- ID (IQ >70)	1797 (98.1)	34 (1.9)	
ID (IQ <70)	653 (93.8)	43 (6.2)	
	**No. (Mean, SD)**		
Full Scale IQ Score	2450 (84.9, 25.7)	77 (66.2, 26.8)	<.001
**Adaptive Functioning**			
Adaptive Behavior Composite Score	3531 (68.1, 17.5)	151 (55.3, 20.7)	<.001
Motor Skills Standard Score	2171 (82.3, 18.4)	94 (73.0, 21.5)	<.001
**ASD Severity**			
ADOS Calibrated Severity Score	3153 (7.1, 1.9)	119 (7.4, 1.8)	0.04

Abbreviations: SD, standard deviation, PPVT, Peabody Picture Vocabulary Test; ADOS, Autism Diagnostic Observation Schedule.

a Genetic Collaborative Samples (AGRE, SSC, and AC) combined.

Values may not add up to total due to missing data.

**Table 6:** Logistic Regression Modeling the Odds of an Epilepsy Diagnosis by Demographic and Clinical Characteristics, Combined Genetic Collaborative Sample^a^.

	Model 1: Unadjusted		Model 2: Adjusted for FSIQ		Model 3: Fully Adjusted	
Characteristic	OR (95% CI)	p-value	OR (95% CI)	p-value	OR (95% CI)	p-value
Age						
9 years and younger	1.00 [Reference]		1.00 [Reference]		1.00 [Reference]	
10 years and older	3.05 (2.29–4.06)	<.001	2.40 (1.51–3.82)	<.001	2.35 (1.42–3.88)	<.001
Gender						
Male	1.00 [Reference]		1.00 [Reference]		1.00 [Reference]	
Female	1.86 (1.35–2.56)	<.001	1.43 (0.82–2.49)	0.21	1.36 (0.77–2.43)	0.29
Cognitive Ability						
Full Scale IQ Score	0.51 (0.4 1–0.63)	<.001	0.51 (0.41–0.63)	<.001	0.53 (0.39–0.73)	<.001
Adaptive Functioning						
Adaptive Behavior Composite Score	0.52 (0.45–0.61)	<.001	0.80 (0.58–1.10)	0.17	0.98 (0.70–1.37)	0.89
Language						
Meaningful Use of Single Words, Two-Word Phrases, or Three-Word Phrases	1.00 [Reference]		1.00 [Reference]		1.00 [Reference]	
Fewer than 5 Words	2.00 (1.39–2.87)	<.001	0.75 (0.27–2.05)	0.57	0.75 (0.27–2.13)	0.59
Developmental Regression						
No Loss of Language or Skills	1.00 [Reference]		1.00 [Reference]		1.00 [Reference]	
Loss of any Language or Skills	1.93 (1.45–2.57)	<.001	1.05 (0.64–1.72)	0.86	1.14 (0.69–1.89)	0.60

Abbreviations: OR, odds ratio; CI, confidence interval; FSIQ, full scale IQ score.

a Genetic Collaborative Samples (AGRE, SSC, and AC) combined.

Model 1: Individual models for each variable.

Model 2: Individual models for each variable, adjusted for full scale IQ score only.

Model 3: Single model adjusted for all variables.

Odds ratios for full scale IQ score and adaptive behavior composite score represent the odds of epilepsy for a one standard deviation increase.

In the NSCH, during the phone-based interview, parents were asked if they had ever been told by a doctor or other health care provider that their child had “epilepsy or seizure disorder.” Children reported to be currently or ever diagnosed with epilepsy were classified as having epilepsy.

### Clinical Characteristics

Clinical characteristics were available for subjects in the genetic collaborative samples (Table 1).

#### Cognitive ability

Cognitive ability was measured via standardized intelligence tests, each of which provided an intelligence quotient (IQ). IQ data were available for a subset of AGRE participants (n = 469) who completed the *Stanford Binet Intelligence Scales, 5th Edition* [33]. IQ data were available for all SSC participants, the majority (n = 1632) of whom completed the *Differential Ability Scales, 2nd Edition (DAS-II)* [34]; a minority completed other cognitive assessments. IQ data were available for a subset of AC participants (n = 273) derived from a variety of intelligence tests including the *Mullen Scales of Early Learning* [35], the *Wechsler Abbreviated Scale of Intelligence (WASI)* [36], the *Wechsler Preschool and Primary Scale of Intelligence, Third Edition (WPPSI-III)* [37], and the *DAS-II*. Full-scale IQ scores were used to create a dichotomous Intellectual Disability (ID) variable defined as IQ at or below 70 (ID) versus above 70 (not ID).

#### Adaptive functioning

Adaptive functioning was measured by the Vineland Adaptive Behavior Scales, Second Edition (Vineland-II) [38], a valid and reliable measure of adaptive functioning. The Adaptive Behavior Composite score is a summary score derived by adding the standard scores for each domain (communication, daily living skills, socialization, and motor skills). The Motor Skills standard score is derived from the motor skills domain, which is comprised of two subdomains: gross and fine motor skills. This score was available for subjects less than seven years of age.

#### Developmental regression

A history of developmental regression was measured by parent response to various questions on the ADI-R. Detailed descriptions of the items can be found in the ADI-R manual [29]. We created a composite variable for any regression, defined as a loss of any previously acquired communication or social skill. This was based on all items from the ADI-R pertaining to loss of language or other skills. We also examined two specific items, “loss of any language” and “loss of any skills.”

#### Language

Language was measured by parent report response to an ADI-R question regarding the child’s current overall use of language and by the *Peabody Picture Vocabulary Test (PPVT)* [39], which measures receptive one-word vocabulary. PPVT data were available for 1,277 AGRE, 1,748 SSC, and 337 AC participants.

#### ASD severity

ASD severity was measured by the ADOS Calibrated Severity score, a severity metric created by Gotham, Pickles, and Lord [40] that takes into account age and language level and is based on raw total scores of the Autism Diagnostic Observation Schedule (ADOS) [41]. The Calibrated Severity score was available for genetic collaborative participants administered ADOS modules 1–3, as it has not been devised for module 4.

### Statistical Methods

Statistical analyses were conducted using SAS software, version 9.3. Bivariate analyses were conducted to compare individuals with and without epilepsy on demographic and clinical characteristics. Chi-square and t-test p-values were calculated when appropriate. Statistical significance was evaluated using 2-sided tests at a 0.05 alpha-level. For the NSCH, we computed the weighted prevalence of epilepsy in the entire sample of children with ASD and in subgroups. Using proc survey weights in SAS, the survey weights adjust the survey responses to reflect characteristics of the non-institutionalized population of children in the US. We report unweighted sample sizes, weighted percentages, and weighted 95% confidence intervals for estimated rates.

Logistic regression was used to examine the association between epilepsy and demographic and clinical characteristics among genetic collaborative study participants. We report odds ratios and 95% confidence intervals. Cases with missing values were excluded. We fit separate models for each variable (model 1), separate models for each variable adjusted for IQ score (model 2), and a model with all of the predictors entered simultaneously (model 3). Model diagnostics performed on the final multivariate model (model 3) included the removal of outliers, checking for normal distribution of continuous predictors, checking for over-dispersion, and testing model fit using the Hosmer-Lemeshow Test. The Vineland Motor Skills standard score and ADOS Calibrated Severity score were not included in the regression models because of the small sample size for these measures.

## Results

### Sample Characteristics

The total sample size from all four studies combined was 5,815 participants with ASD, of whom 289 had co-occurring epilepsy. The majority of total participants were male, ranging from 80.3% male in AGRE to 86.4% male in SSC, and the majority were between the ages of 4 and 12 years (**Table 2**). Most participants were White, ranging from 66.9% White in the NSCH to 85.9% White in the AC. Among genetic collaborative study total participants for whom IQ data were available, 33.3% of AGRE, 28.8% of SSC, and 15.3% of AC participants had intellectual disability (ID).

### Occurrence of Epilepsy

The distribution of epilepsy in the AGRE, SSC, and AC studies was 5.3% (n = 120), 2.9% (n = 51), and 6.7% (n = 30), respectively with a combined frequency of 4.5% (n = 201) (**Table 3**). In the population-based sample, the NSCH, the prevalence of epilepsy was 12.5% (n = 88).

### Clinical Characteristics of Individuals with Asd and Epilepsy

**Figure 1:**
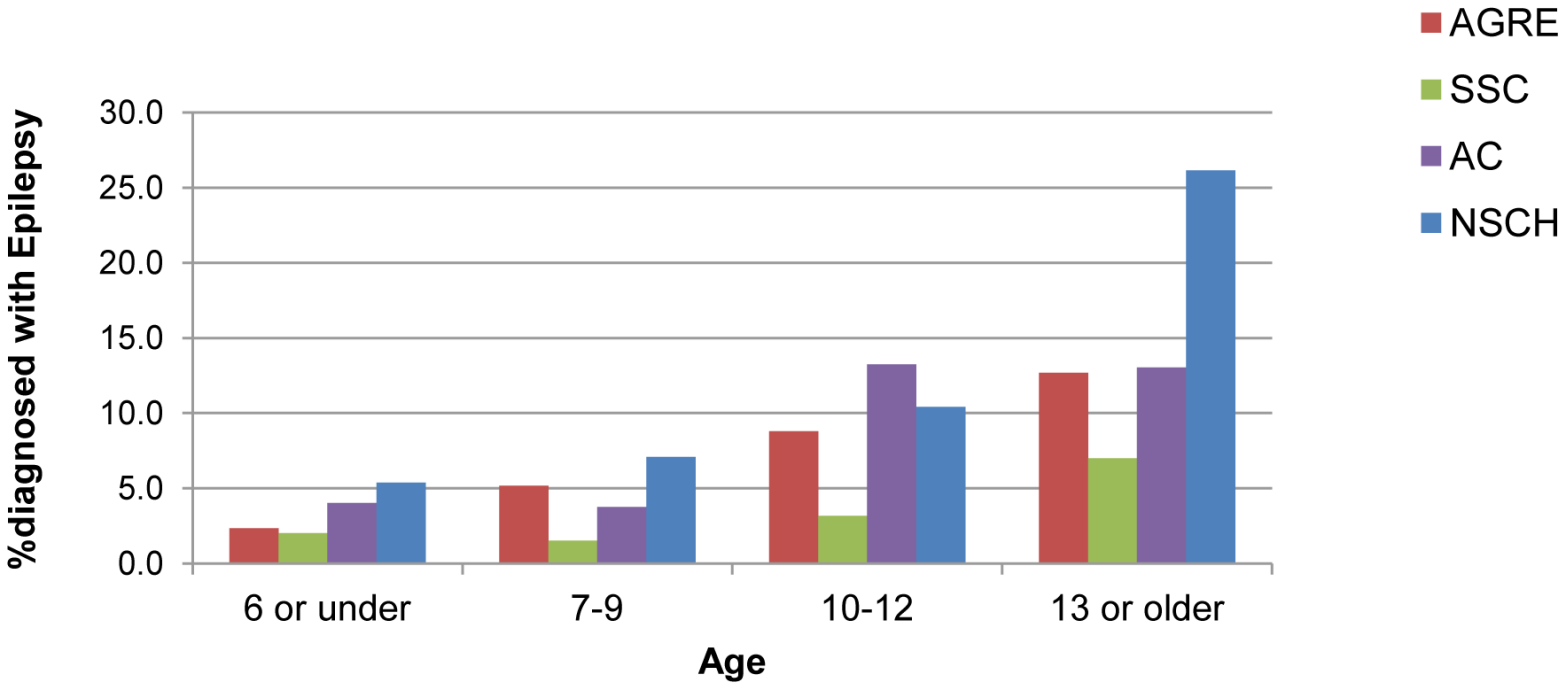
Prevalence of Epilepsy by Age among Individuals with Autism Spectrum Disorder, All Studies. The percentage of children with epilepsy by age group by study sample. The prevalence is higher in older children than younger children. Abbreviations: AGRE, the Autism Genetic Resource Exchange; SSC, the Simons Simplex Collection; AC, the Autism Consortium; NSCH, 2007 the National Survey of Children’s Health.

Findings comparing individuals with ASD with and without epilepsy are presented. Individual-level data from the genetic collaborative samples were combined. Results from each of the genetic collaborative samples can be found in Tables S1–S9 in Supporting Information S1. As shown in **Table 4** and **Figure 1**, epilepsy was more prevalent in older children. Among children aged 13 or older, 10.3% had epilepsy in the combined genetic collaborative sample and 26.2% had epilepsy in the NSCH. Epilepsy was more prevalent in females in the combined genetic collaborative sample; 7% of females had epilepsy as compared to 3.9% of males (p<.001). In all of the genetic collaborative samples the prevalence of epilepsy was greater in females, but this difference was only statistically significant in the AGRE study (Table S2 in Supporting Information S1). In the NSCH, there were no gender differences in epilepsy prevalence. We also found that among both males and females epilepsy rates increased significantly with age.

**Table 5** presents comparisons of clinical characteristics in children with ASD with and without epilepsy from the combined genetic collaborative sample. Children with a history of developmental regression were significantly more likely to have epilepsy (6.7% of children with definite loss of language or skills had epilepsy, as compared to 3.6% without loss, p<.001). With regard to language, epilepsy was more prevalent in children with fewer than 5 words and children with epilepsy had a significantly lower mean PPVT score (67.8 vs. 85.9, p<.001). Epilepsy was associated with significantly lower cognitive ability, as evidenced by a lower mean IQ score in the epilepsy group (66.2 vs. 84.9, p<.001) and a greater prevalence of epilepsy in children with ID (6.2% vs. 1.9%, p<.001). Individuals with epilepsy also had poorer adaptive functioning, as evidenced by significantly lower mean Adaptive Behavior Composite score and Motor Skills standard score. Children with epilepsy also had a significantly higher mean ADOS Calibrated Severity score (7.4 vs. 7.1, p = 0.04), indicating more severe ASD symptoms.

**Table 6** presents logistic regression analyses modeling the odds of epilepsy by demographic and clinical characteristics for participants from the genetic collaborative samples. In the unadjusted models (model 1), all of the characteristics were significantly associated with epilepsy. In the models adjusted for IQ (model 2), age was the only variable that remained associated with epilepsy. In the multivariate model that adjusted for all of the variables (model 3), age and full scale IQ score were the only variables that significantly increased risk of epilepsy. Controlling for all other variables in the model, individuals age 10 or older had 2.35 times the odds of being diagnosed with epilepsy (p<.001) and for a one standard deviation increase in full scale IQ, the odds of having epilepsy decreased by 47% (p<.001).

## Discussion

This is among the largest studies to date of children with ASD and co-occurring epilepsy. Our sample includes 5,815 participants with ASD, 289 of whom had co-morbid epilepsy. Using statistical modeling in this well-powered sample of patients we have made several important observations about a contemporary group of individuals with ASD and epilepsy. We identified several correlates of epilepsy in children with ASD including older age, lower cognitive and adaptive functioning, poorer language skills, a history of developmental regression, and more severe ASD symptoms. Through multivariate logistic regression we found that only age and cognitive ability were independent predictors of epilepsy.

The average prevalence of epilepsy among children aged 2 to 17 years in our population-based sample, the NSCH, was 12.5%. This estimate is comparable to a recent report of a 15.5% rate of epilepsy in another population-based sample of children with ASD [42]. While the prevalence was 10% or lower in children under 13 years of age, by adolescence it reached 26.2%. Therefore, the best estimate of the cumulative prevalence of epilepsy in ASD through 17 years of age is 26%. Our study replicates findings from prior studies that have followed children with ASD into adolescence/early adulthood and reported epilepsy prevalence rates from 22% to 38% [14,15,21].

Population-based samples like the NSCH can provide accurate estimates of the prevalence of epilepsy in the general population of children with ASD by random sampling, which reduces sampling bias and improves generalizability. However, these samples often have smaller numbers of ASD cases and lack detailed phenotypic information on study participants. In contrast, samples from genetic collaboratives are less likely to be representative of the greater ASD population due to specific inclusion and exclusion criteria; however, these datasets are often large, include carefully confirmed cases of ASD, and provide rich phenotypic information collected with modern assessment tools. In the present study, we make use of both a population-based sample and several genetic collaborative samples allowing for an estimate of the prevalence of epilepsy in ASD and an examination of important clinical correlates of ASD and epilepsy. The average prevalence of epilepsy found in the combined genetic collaborative sample of 4.5% and the cumulative estimate of 10.3% in children aged 13 and older was considerably lower than the prevalence in the NSCH. This likely reflects the specific inclusion and exclusion criteria of these samples, in particular, the tendency to recruit children with higher-functioning ASD and to exclude children with certain disorders and other conditions (i.e. some known genetic syndromes) associated with epilepsy.

Additional strengths of our study are the large sample size and detailed and standardized assessments of clinical correlates using modern, reliable measures. The large sample size allowed us to use regression modeling to examine the association between epilepsy and various characteristics in a multivariate model. Because of their small sample size, most prior studies have been unable to control for confounders in the examination of risk factors for epilepsy in ASD. In particular, there has been a need for an examination of gender and epilepsy controlling for confounding by cognitive ability [20].

As reported in previous studies [14,15,20,21], individuals with epilepsy in our study had lower cognitive ability and were more likely to have ID. Over half of children with epilepsy in the genetic collaborative samples had ID and the mean IQ score for individuals with ASD and co-morbid epilepsy was 66.2. Although our findings with regard to IQ and epilepsy replicate those reported in prior studies, our results are important given that the IQ distribution of children with ASD has shifted considerably in the last few years, as many more children with ASD without ID are being identified [43]. The relationship between ASD, epilepsy and IQ is complex, and some investigators believe that the association between autism and epilepsy is primarily driven by the presence of ID [44]. van Eeghen et al. [45] found a strong inverse association between autistic traits and IQ in persons with epilepsy. They concluded that autistic features appear to be part of the neurocognitive construct of disorders like epilepsy. They also found associations between autistic traits and epilepsy in patients with Tuberous Sclerosis. In addition to IQ, the present study examined other related measures such as adaptive functioning and language skills, which have not been extensively studied.

Our findings with regard to gender were mixed. Females were significantly more likely to have epilepsy in the combined genetic collaborative sample, but males were more likely to have epilepsy in the NSCH (although this difference was not statistically significant). We also found variation in the association between gender and epilepsy in the individual genetic collaborative samples; while all of the samples showed a higher proportion of females with epilepsy, this difference was only statistically significant in the multiplex sample (AGRE). Tuchman [46] found that female gender was not a risk factor for epilepsy after controlling for ID and motor deficit. In contrast, Bolton [15] found that female gender was significantly associated with epilepsy even after adjusting for verbal ability and non-verbal IQ. In the present study, female gender was associated with epilepsy among subjects of the genetic collaborative samples only in the unadjusted logistic regression model; gender was not an independent risk factor for epilepsy after adjusting for IQ. The association between gender and epilepsy is theorized to be due to a greater proportion of females with ASD having low cognitive ability [47], as low IQ is associated with epilepsy. This is supported by our finding that female gender was not associated with increased risk of epilepsy after controlling for IQ. However, this theory cannot be confirmed based on our statistical observations alone. It is also possible that there is a biological mechanism by which females with ASD are at increased risk for both lower IQ and epilepsy. Further research is needed to better understand the association between gender and epilepsy in ASD and to determine if the association differs in multiplex versus simplex families.

Epilepsy onset in persons without autism has been shown to be highest in the first year of life [48,49] and generally shows a bi-modal curve with higher rates in early and later life [50]. In persons with ASD, two peaks of seizure onset have been reported, one in early childhood [9] and one in adolescence and continuing through adulthood [51]. This pattern may be unique to individuals with ASD [5]. We found a higher prevalence of epilepsy in children with ASD of older age, which is expected given that older children have had a longer amount of time to develop epilepsy. The pattern was evident in both males and females with ASD showing that it is independent of gender. This finding is in line with other studies that report the highest epilepsy rates in samples that include adolescents and adults. It is also consistent with studies showing a peak in epilepsy onset in adolescence [52]. A limitation of our study is that the sample included a large number of young participants, and given that this is a cross-sectional study, we cannot be certain that some of the participants will not develop epilepsy at a later time.

Additional limitations should be considered when interpreting the results of this study. First, we relied exclusively on parent report for the diagnosis of epilepsy, which may have resulted in misclassification. For a subset of participants from the genetic collaborative samples (n = 2,525) we were able to cross-validate parent report of epilepsy diagnosis on the ADI-R with assessment of non-febrile seizures based on a medical history interview or questionnaire. We found that children reported by their parent to have epilepsy on the ADI-R were highly likely (95%) to also have a history of non-febrile seizures based on medical history (Table S10 in Supporting Information S1). This provides support for the reliability of the parent-report epilepsy measure. Furthermore, previous studies have used the ADI-R to identify cases of epilepsy in children with ASD [15,53]. In addition, we relied on parent-report of ASD diagnosis in the NSCH. However, a number of studies have utilized maternally-reported diagnosis of ASD and other child health conditions and shown the reliability of these reports [54,55]. In addition, parent report of medical conditions in the NSCH were consistent with those expected by clinical assessment [56] and the NSCH has been used to estimate the prevalence of ASD in the United States [57].

Another limitation is that the genetic collaborative samples were ascertained based on the nature of the pedigrees sought, specific inclusion and exclusion criteria, and the ability of the affected participants to complete the extensive phenotyping batteries. A smaller percentage of participants in the genetic collaborative samples had cognitive abilities in the ID range, as compared to the rate in the general ASD population, recently reported to be 38% [43]. This may in part explain the lower occurrence of epilepsy found in these samples as compared to the NSCH. We did not use the genetic collaborative samples for estimates of epilepsy prevalence given that they are not population-based samples. Instead, we used these samples to examine clinical characteristics that are less likely to be affected by this sampling bias.

There was also some heterogeneity across the genetic collaborative samples in the occurrence and clinical correlates of epilepsy due to differences between the samples. We combined data from the genetic collaborative samples in our main analyses, which may not reflect the findings from the individual samples (see Tables S1–S9 in Supporting Information S1 for results from the individual studies). However, these differences were minor and in general, the results were similar across samples, suggesting that combining individual level data from each of the studies was appropriate.

An additional caveat to our study is missing data. In particular, IQ data were available for only a subset of participants. Genetic study participants for whom we had IQ data had somewhat higher IQ than would be expected in a representative sample of children with ASD (as mentioned above). The subjects used in the multivariate regression models were more likely to be from the SSC sample due to missing IQ data among AC and AGRE participants. As such, the findings may be more applicable to children with ASD from simplex families who meet the specific inclusion criteria of the SSC study. We conducted sensitivity analyses to determine if the results would differ if more participants from the AC and AGRE samples were included in the regression model. When we ran the analyses using PPVT score as a measure cognitive ability (which was available for the majority of genetic collaborative participants), instead of IQ score, the results were the same. Furthermore, when fully adjusted regression models were run separately in each of the genetic collaborative study samples the results were similar to the results from the combined sample (Table S9 in Supporting Information S1). The effect sizes (odds ratios) from the individual studies were similar to the combined sample; however in the AGRE sample gender was a significant predictor of epilepsy and IQ was a non-significant predictor. This is likely to be explained by the small number of AGRE participants with IQ data.

Individuals with ASD and epilepsy are an important subgroup of patients who require specialized medical care and may be of etiological significance to understanding the neurobiology of ASD. It has been suggested that the most common reason for the co-occurrence of ASD and epilepsy is that the same brain pathology causes both disorders [58]. Turk et al. [18] found that children with ASD and epilepsy were more likely to receive a later ASD diagnosis and have additional medical disorders, motor difficulties, developmental delays, and challenging behaviors, compared to children with ASD only. Perhaps most striking, persons with ASD and epilepsy have higher mortality rates [21,59]. The ASD-epilepsy subgroup may also be helpful to genetic research into ASD etiology. Duplications of the 15q11–13 locus or 15q13 copy number variants are frequently associated with ASD and epilepsy, and several key candidate genes are located in these intervals [3]. Recent sequencing studies in autism have identified *de novo* variants in a variety of epilepsy-related genes [60,61,62,63].

### Conclusions

Our findings suggest that epilepsy is a common co-morbid condition in individuals with ASD, occurring in approximately 12% of children with ASD and reaching 26% by adolescence. In a large, contemporary sample of children with ASD, we identified several risk factors for epilepsy including older age, low IQ and adaptive functioning, poor language skills, a history of developmental regression, and more severe ASD symptoms. Through statistical modeling we demonstrated that the most-widely reported factors associated with epilepsy are not predictive after adjusting for IQ. Low IQ is the best clinical predictor of epilepsy in children with ASD. These findings can help guide prognosis and alert clinicians to patients who are at increased risk for epilepsy.

## Supporting Information

Supporting Information S1 **Supporting Tables.** **Table S1** Epilepsy Diagnosis by Age among Individuals with Autism Spectrum Disorder, Genetic Collaborative Samples. The prevalence of epilepsy was significantly higher in older children in all of the genetic collaborative samples. **Table S2** Epilepsy Diagnosis by Gender among Individuals with Autism Spectrum Disorder, Genetic Collaborative Samples. The prevalence of epilepsy was higher in females with ASD in all of the genetic collaborative samples, but this difference only reached statistical significance in the AGRE sample. **Table S3** Epilepsy Diagnosis by History of Developmental Regression among Individuals with Autism Spectrum Disorder, Genetic Collaborative Samples. The prevalence of epilepsy was higher in individuals with a history of developmental regression in all of the genetic collaborative samples. **Table S4** Epilepsy Diagnosis by Language among Individuals with Autism Spectrum Disorder, Genetic Collaborative Samples. The prevalence of epilepsy was significantly higher in individuals with fewer than 5 words in all of the genetic collaborative samples. **Table S5** Epilepsy Diagnosis by Cognitive Ability among Individuals with Autism Spectrum Disorder, Genetic Collaborative Samples. Individuals with epilepsy had significantly lower cognitive ability in all of the genetic collaborative samples. **Table S6** Epilepsy Diagnosis by Intellectual Disability among Individuals with Autism Spectrum Disorder, Genetic Collaborative Samples. The prevalence of epilepsy was higher in individuals with intellectual disability in all of the genetic collaborative samples. **Table S7** Epilepsy Diagnosis by Adaptive Functioning among Individuals with Autism Spectrum Disorder, Genetic Collaborative Samples. Individuals with epilepsy had significantly lower adaptive functioning in all of the genetic collaborative samples. **Table S8** Epilepsy Diagnosis by Autism Severity among Individuals with Autism Spectrum Disorder, Genetic Collaborative Samples. Individuals with epilepsy had higher mean ADOS Calibrated Severity scores in all of the genetic collaborative samples. **Table S9** Logistic Regression Modeling the Odds of an Epilepsy Diagnosis by Demographic and Clinical Characteristics, Individual Genetic Collaborative Samples. Logistic regression model findings were similar in participants of the individual genetic collaborative samples to the results from the combined sample. **Table S10** Cross-Validation of Parent Report Epilepsy Diagnosis on the ADI-R with Report of Non-Febrile Seizures based on Medical History, Subset of Genetic Collaborative Study Participants (n = 2,525). There was good agreement between parent report of epilepsy diagnosis on the ADI-R and medical history.
(DOCX)
