# Supplementary material for: Clinical Characteristics of Children with Autism Spectrum Disorder and Co-Occurring Epilepsy
Source: PLoS One. 2013 Jul 4;8(7):e67797. doi: 10.1371/journal.pone.0067797 (PMC3701630; doi:10.1371/journal.pone.0067797)
Supplement: Supporting Information S1 — Supporting Tables. Table S1 Epilepsy Diagnosis by Age among Individuals with Autism Spectrum Disorder, Genetic Collaborative Samples. The prevalence of epilepsy was significantly higher in older children in all of the genetic collaborative samples. Table S2 Epilepsy Diagnosis by Gender among Individuals with Autism Spectrum Disorder, Genetic Collaborative Samples. The prevalence of epilepsy was higher in females with ASD in all of the genetic collaborative samples, but this difference only reached statistical significance in the AGRE sample. Table S3 Epilepsy Diagnosis by History of Developmental Regression among Individuals with Autism Spectrum Disorder, Genetic Collaborative Samples. The prevalence of epilepsy was higher in individuals with a history of developmental regression in all of the genetic collaborative samples. Table S4 Epilepsy Diagnosis by Language among Individuals with Autism Spectrum Disorder, Genetic Collaborative Samples. The prevalence of epilepsy was significantly higher in individuals with fewer than 5 words in all of the genetic collaborative samples. Table S5 Epilepsy Diagnosis by Cognitive Ability among Individuals with Autism Spectrum Disorder, Genetic Collaborative Samples. Individuals with epilepsy had significantly lower cognitive ability in all of the genetic collaborative samples. Table S6 Epilepsy Diagnosis by Intellectual Disability among Individuals with Autism Spectrum Disorder, Genetic Collaborative Samples. The prevalence of epilepsy was higher in individuals with intellectual disability in all of the genetic collaborative samples. Table S7 Epilepsy Diagnosis by Adaptive Functioning among Individuals with Autism Spectrum Disorder, Genetic Collaborative Samples. Individuals with epilepsy had significantly lower adaptive functioning in all of the genetic collaborative samples. Table S8 Epilepsy Diagnosis by Autism Severity among Individuals with Autism Spectrum Disorder, Genetic Collaborative Samples. Individuals with epilepsy had [file pone.0067797.s001.docx]

**Supporting Information S1: Supporting Tables**

| Table S1. Epilepsy Diagnosis by Age among Individuals with Autism Spectrum Disorder, Genetic Collaborative Samples | | | | | | | | | |
| --- | --- | --- | --- | --- | --- | --- | --- | --- | --- |
|  | **AGRE** | | | **SSC** | | | **AC** | | |
|  | **Never**  **Diagnosed**  **with Epilepsy** | **Diagnosed**  **with Epilepsy** |  | **Never**  **Diagnosed**  **with Epilepsy** | **Diagnosed**  **with Epilepsy** |  | **Never**  **Diagnosed**  **with Epilepsy** | **Diagnosed**  **with Epilepsy** |  |
|  | **No. (%)** | | **p-value** | **No. (%)** | | **p-value** | **No. (%)** | | **p-value** |
| Age (years) |  |  | <.001 |  |  | <.001 |  |  | 0.006 |
| 6 or under | 1039 (97.7) | 25 (2.4) |  | 632 (98.0) | 13 (2.0) |  | 190 (96.0) | 8 (4.0) |  |
| 7-9 | 586 (94.8) | 32 (5.2) |  | 514 (98.5) | 8 (1.5) |  | 102 (96.2) | 4 (3.8) |  |
| 10-12 | 280 (91.2) | 27 (8.8) |  | 337 (96.8) | 11 (3.2) |  | 59 (86.8) | 9 (13.2) |  |
| 13 or older | 248 (87.3) | 36 (12.7) |  | 252 (93.0) | 19 (7.0) |  | 60 (87.0) | 9 (13.0) |  |

Abbreviations: AGRE, the Autism Genetic Resource Exchange; SSC, the Simons Simplex Collection; AC, the Autism Consortium.

| Table S2. Epilepsy Diagnosis by Gender among Individuals with Autism Spectrum Disorder, Genetic Collaborative Samples | | | | | | | | | | |
| --- | --- | --- | --- | --- | --- | --- | --- | --- | --- | --- |
|  | **AGRE** | | | | **SSC** | | | **AC** | | |
|  | **Never Diagnosed**  **with Epilepsy** | | **Diagnosed**  **with Epilepsy** |  | **Never Diagnosed**  **With Epilepsy** | **Diagnosed**  **with Epilepsy** |  | **Never Diagnosed**  **With Epilepsy** | **Diagnosed**  **with Epilepsy** |  |
|  | **No. (%)** | | | **p-value** | **No. (%)** | | **p-value** | **No. (%)** | | **p-value** |
| Gender |  |  | |  |  |  |  |  |  |  |
| Male | 1745 (95.5) | 83 (4.5) | | .001 | 1500 (97.3) | 42 (2.7) | 0.40 | 343 (94.0) | 22 (6.0) | 0.26 |
| Female | 408 (91.7) | 37 (8.3) | |  | 235 (96.3) | 9 (3.7) |  | 77 (90.6) | 8 (9.4) |  |

Abbreviations: AGRE, the Autism Genetic Resource Exchange; SSC, the Simons Simplex Collection; AC, the Autism Consortium.

| Table S3. Epilepsy Diagnosis by History of Developmental Regression among Individuals with Autism Spectrum Disorder, Genetic Collaborative Samples | | | | | | | | | |
| --- | --- | --- | --- | --- | --- | --- | --- | --- | --- |
|  | **AGRE** | | | **SSC** | | | **AC** | | |
|  | **Never Diagnosed**  **with Epilepsy** | **Diagnosed**  **with Epilepsy** |  | **Never Diagnosed**  **with Epilepsy** | **Diagnosed**  **64093ldren from AGRE and ng data on seizures, and 61 children from with Epilepsy** |  | **Never**  **Diagnosed**  **with Epilepsy** | **Diagnosed**  **with Epilepsy** |  |
|  | **No. (%)** | | **p-value** | **No. (%)** | | **p-value** | **No. (%)** | | **p-value** |
| Any Regression |  |  | <.001 |  |  | 0.25 |  |  | <.001 |
| No Definite Loss | 1517 (95.8) | 66 (4.2) |  | 1251 (97.4) | 33 (2.6) |  | 330 (95.7) | 15 (4.3) |  |
| Definite Loss | 636 (92.2) | 54 (7.8) |  | 484 (96.4) | 18 (3.6) |  | 90 (85.7) | 15 (14.3) |  |
| Loss of Any Language |  |  | 0.11 |  |  | 0.03 |  |  | 0.04 |
| No | 1128 (96.6) | 40 (3.4) |  | 1432 (97.6) | 35 (2.4) |  | 354 (94.4) | 21 (5.6) |  |
| Yes | 260 (94.6) | 15 (5.5) |  | 290 (95.4) | 14 (4.6) |  | 65 (87.8) | 9 (12.2) |  |
| Loss of Skills |  |  | 0.05 |  |  | 0.47 |  |  | <.001 |
| No Consistent Loss | 1007 (96.9) | 32 (3.1) |  | 1325 (97.4) | 36 (2.6) |  | 351 (96.2) | 14 (3.8) |  |
| Probable Loss | 42 (97.7) | 1 (2.3) |  | 64 (94.1) | 4 (5.9) |  | 21 (77.8) | 6 (22.2) |  |
| Definite Loss | 333 (94.1) | 21 (5.9) |  | 341 (97.2) | 10 (2.8) |  | 38 (80.8) | 9 (19.2) |  |

Abbreviations: AGRE, the Autism Genetic Resource Exchange; SSC, the Simons Simplex Collection; AC, the Autism Consortium.

| Table S4. Epilepsy Diagnosis by Language among Individuals with Autism Spectrum Disorder, Genetic Collaborative Samples | | | | | | | | | | |
| --- | --- | --- | --- | --- | --- | --- | --- | --- | --- | --- |
|  | **AGRE** | | | | **SSC** | | | **AC** | | |
|  | **Never**  **Diagnosed**  **with Epilepsy** | **Diagnosed**  **with Epilepsy** | |  | **Never Diagnosed**  **with Epilepsy** | **Diagnosed**  **with Epilepsy** |  | **Never**  **Diagnosed**  **with Epilepsy** | **Diagnosed**  **with Epilepsy** |  |
|  | **No. (%)** | | | **p-value** | **No. (%)** | | **p-value** | **No. (%)** | | **p-value** |
| Overall Level of Language |  | |  | 0.03 |  |  | 0.02 |  |  | 0.007 |
| Meaningful Use of Phrases | 1453 (95.7) | | 66 (4.3) |  | 1574 (97.5) | 40 (2.5) |  | 339 (95.2) | 17 (4.8) |  |
| Fewer than 5 Words | 374 (93.0) | | 28 (7.0) |  | 48 (92.3) | 4 (7.7) |  | 41 (85.4) | 7 (14.6) |  |

Abbreviations: AGRE, the Autism Genetic Resource Exchange; SSC, the Simons Simplex Collection; AC, the Autism Consortium.

| Table S5. Epilepsy Diagnosis by Cognitive Ability among Individuals with Autism Spectrum Disorder, Genetic Collaborative Samples | | | |
| --- | --- | --- | --- |
|  | **Never Diagnosed**  **with Epilepsy** | **Diagnosed**  **with Epilepsy** |  |
|  | **No. (Mean, SD)** | | **p-value** |
| **AGRE** |  |  |  |
| Full Scale IQ (n = 469) | 456 (82.6, 23.6) | 13 (75.2, 30.4) | 0.27 |
| PPVT Standard Score  (n = 1277) | 1233 (83.7, 26.7) | 44 (73.1, 32.9) | 0.04 |
| **SSC** |  |  |  |
| Full Scale IQ (n = 1785) | 1734 (84.1, 26.5) | 51 (61.3, 26.5) | <.001 |
| PPVT Standard Score  (n = 1748) | 1699 (86.8, 28.4) | 49 (64.7, 30.6) | <.001 |
| **AC** |  |  |  |
| Full Scale IQ (n = 273) | 260 (94.3, 21.1) | 13 (76.7, 16.2) | 0.003 |
| PPVT Standard Score  (n = 337) | 320 (89.6, 28.9) | 17 (63.1, 27.4) | <.001 |

Abbreviations: SD, standard deviation; AGRE, the Autism Genetic Resource Exchange; SSC, the Simons Simplex Collection; PPVT, Peabody Picture Vocabulary Test.

| Table S6. Epilepsy Diagnosis by Intellectual Disability among Individuals with Autism Spectrum Disorder, Genetic Collaborative Samples | | | | | | | | | |
| --- | --- | --- | --- | --- | --- | --- | --- | --- | --- |
|  | **AGRE** | | | **SSC** | | | **AC** | | |
|  | **Never Diagnosed**  **with Epilepsy** | **Diagnosed with Epilepsy** |  | **Never Diagnosed**  **with Epilepsy** | **Diagnosed with Epilepsy** |  | **Never Diagnosed**  **with Epilepsy** | **Diagnosed with Epilepsy** |  |
|  | **No (%)** | | **p-value** | **No (%)** | | **p-value** | **No (%)** | | **p-value** |
| Intellectual Disability (ID) |  |  | 0.08 |  |  | <.001 |  |  | 0.02 |
| Non- ID (IQ > 70) | 314 (98.1) | 6 (1.9) |  | 1259 (98.4) | 20 (1.6) |  | 224 (96.6) | 8 (3.4) |  |
| ID (IQ < 70) | 143 (95.3) | 7 (4.7) |  | 475 (93.9) | 31 (6.1) |  | 36 (87.8) | 5 (12.2) |  |

Abbreviations: AGRE, the Autism Genetic Resource Exchange; SSC, the Simons Simplex Collection; AC, the Autism Consortium.

| Table S7. Epilepsy Diagnosis by Adaptive Functioning among Individuals with Autism Spectrum Disorder, Genetic Collaborative Samples | | | | | | | | | |
| --- | --- | --- | --- | --- | --- | --- | --- | --- | --- |
|  | **AGRE** | | | **SSC** | | | **AC** | | |
|  | **Never**  **Diagnosed**  **with Epilepsy** | **Diagnosed**  **with Epilepsy** |  | **Never**  **Diagnosed**  **with Epilepsy** | **Diagnosed**  **with Epilepsy** |  | **Never Diagnosed**  **with Epilepsy** | **Diagnosed**  **with Epilepsy** |  |
|  | **No. (Mean, SD)** | | **p-value** | **No. (Mean, SD)** | | **p-value** | **No. (Mean, SD)** | | **p-value** |
| Adaptive Behavior Composite Score | 1439 (58.9, 19.0) | 74 (45.9, 19.9) | <.001 | 1735 (74.5, 11.5) | 51 (65.9, 12.0) | <.001 | 357 (74.5, 19.2) | 26 (61.3, 23.8) | 0.001 |
| Motor Skills Standard Score | 1369 (82.2, 20.9) | 70 (72.6, 23.6) | <.001 | 631 (82.8, 12.6) | 14 (76.5, 11.0) | 0.06 | 171 (80.7, 14.0) | 10 (70.8, 17.8) | 0.03 |

Abbreviations: SD, standard deviation; AGRE, the Autism Genetic Resource Exchange; SSC, the Simons Simplex Collection; AC, the Autism Consortium.

| Table S8. Epilepsy Diagnosis by Autism Severity among Individuals with Autism Spectrum Disorder, Genetic Collaborative Samples | | | | | | | | | |
| --- | --- | --- | --- | --- | --- | --- | --- | --- | --- |
|  | **AGRE** | | | **SSC** | | | **AC** | | |
|  | **Never**  **Diagnosed**  **with Epilepsy** | **Diagnosed**  **with Epilepsy** |  | **Never**  **Diagnosed**  **with Epilepsy** | **Diagnosed**  **with Epilepsy** |  | **Never Diagnosed**  **with Epilepsy** | **Diagnosed**  **with Epilepsy** |  |
|  | **No. (Mean, SD)** | | **p-value** | **No. (Mean, SD)** | | **p-value** | **No. (Mean, SD)** | | **p-value** |
| ADOS Calibrated Severity Score | 1200 (6.8, 1.8) | 58 (7.2, 1.8) | 0.07 | 1679 (7.4, 1.7) | 50 (7.8, 1.7) | 0.08 | 274 (6.8, 2.6) | 11 (7.1, 2.3) | 0.70 |

Abbreviations: SD, standard deviation; AGRE, the Autism Genetic Resource Exchange; SSC, the Simons Simplex Collection; AC, the Autism Consortium; ADOS, Autism Diagnostic Observation Schedule.

| Table S9. Logistic Regression Modeling the Odds of an Epilepsy Diagnosis by Demographic and Clinical Characteristics, Individual Genetic Collaborative Samples | | | | | | |
| --- | --- | --- | --- | --- | --- | --- |
|  | **AGRE**  **(n = 430)** | | **SSC**  **(n = 1785)** | | **AC**  **(n = 260)** | |
| **Characteristic** | **OR (95% CI)** | **p-value** | **OR (95% CI)** | **p-value** | **OR (95% CI)** | **p-value** |
| Age |  |  |  |  |  |  |
| 9 years and younger | 1.00 [Reference] |  | 1.00 [Reference] |  | 1.00 [Reference] |  |
| 10 years and older | 1.70 (1.02-2.86) | 0.04 | 1.52 (1.15-2.02) | 0.004 | 2.07 (1.14-3.77) | 0.02 |
| Gender |  |  |  |  |  |  |
| Male | 1.00 [Reference] |  | 1.00 [Reference] |  | 1.00 [Reference] |  |
| Female | 3.52 (1.05-11.78) | 0.04 | 1.05 (0.49-2.23) | 0.91 | 0.75 (0.15-3.82) | 0.73 |
| Cognitive Ability |  |  |  |  |  |  |
| Full Scale IQ Score | 0.80 (0.41-1.57) | 0.53 | 0.47 (0.31-0.71) | 0.004 | 0.35 (0.16-0.74) | 0.01 |
| Adaptive Functioning |  |  |  |  |  |  |
| Adaptive Behavior Composite Score | 0.87 (0.43-1.77) | 0.70 | 0.96 (0.61-1.53) | 0.88 | 0.87 (0.40-1.90) | 0.72 |
| Language |  |  |  |  |  |  |
| Meaningful Use of Single Words, Two-Word Phrases,  or Three-Word Phrases | 1.00 [Reference] |  | 1.00 [Reference] |  | 1.00 [Reference] |  |
| Fewer than 5 Words | 2.24 (0.21-24.12) | 0.51 | 0.72 (0.21-2.42) | 0.59 | *Not estimated* | 0.98 |
| Developmental Regression |  |  |  |  |  |  |
| No Loss of Language or Skills | 1.00 [Reference] |  | 1.00 [Reference] |  | 1.00 [Reference] |  |
| Loss of any Language  or Skills | 1.52 (0.46-5.04) | 0.49 | 0.92 (0.49-1.71) | 0.78 | 2.39 (0.64-8.86) | 0.19 |

Abbreviations: OR, odds ratio; CI, confidence interval; FSIQ, full scale IQ score; AGRE, the Autism Genetic Resource Exchange; SSC, the Simons Simplex Collection; AC, the Autism Consortium.

Model is adjusted for all variables.

Odds ratios for full scale IQ score and adaptive behavior composite score represent the odds of epilepsy for a one standard deviation increase.

| Table S10. Cross-Validation of Parent Report Epilepsy Diagnosis on the ADI-R with Report of Non-Febrile Seizures based on Medical History, Subset of Genetic Collaborative Study Participants (n=2,525) | | |
| --- | --- | --- |
|  | **Parent Report of Epilepsy Diagnosis on ADI-R** | |
| **Parent Report of Non-Febrile Seizures on Medical History** | Never Diagnosed with Epilepsy | Diagnosed with Epilepsy |
|  | **No. (%)** | |
| No Non-Febrile Seizures | 2372 (97.8) | 5 (5.0) |
| One or More Non-Febrile Seizures | 53 (2.2) | 95 (95.0) |

Abbreviations: ADI-R, Autism Diagnostic Interview- Revised (ADI-R).
